# Research on the Optimization and Regulation Mechanism of Waterproofing, Impermeability, and Water Vapor Transmission Property of Mortar Based on Different Modifiers

**DOI:** 10.3390/ma18102363

**Published:** 2025-05-19

**Authors:** Zelei Li, Chuanchuan Guo, Lanlan Xu, Ru Wang

**Affiliations:** 1 Key Laboratory of Advanced Civil Engineering Materials of Ministry of Education, School of Materials Science and Engineering, Tongji University, Shanghai 201804, China; lizelei2000@163.com (Z.L.); 2331527@tongji.edu.cn (L.X.); 2 School of Chemistry and Chemical Engineering, Henan Institute of Science and Technology, Xinxiang 453003, China; guochchch@126.com

**Keywords:** cement mortar, capillary water absorption, impermeability, water vapor transmission property, porosity and pore characteristics, water contact angle, low-field nuclear magnetic resonance

## Abstract

It is challenging for mortar to simultaneously enhance the transmission property of water vapor while maintaining excellent waterproofness and impermeability. However, in some applications, both are necessary. Therefore, three different kinds of modifiers, i.e., cementitious capillary crystalline waterproof materials (XYPEX), γ-methacryloxy-propyl-trimethoxy-silane (KH570), and styrene-butadiene rubber latex (SB), are employed to explore how modified mortar can possess excellent waterproofness, impermeability, and the water vapor transmission property simultaneously. Combining characterization techniques, the influencing factors of these three properties are studied. The results indicate that XYPEX promotes the formation of hydration products within pores, improves waterproofness and impermeability, but decreases the water vapor transmission property. KH570 introduces numerous pores ranging from 0.1 to 5 micrometers and enhances the hydrophobicity of mortar; at 1.25% and 2.5% contents, the modified mortar exhibits excellent waterproofness and water vapor transmission property but poor impermeability. SB introduces numerous air pores and forms polymer films; at 20% content, the modified mortar exhibits excellent waterproofness and water vapor transmission property, with impermeability remaining unchanged, making SB a favorable modifier that combines these three properties. Finally, the mechanisms of these three properties are discussed, which provides a theoretical reference for the control of mortar’s waterproofing, impermeability, and water vapor transmission. The selection of modifiers is based on the actual performance requirements.

## 1. Introduction

Having properties of both waterproofness and water vapor transmission is necessary in some applications. When there is a pressure difference of water vapor between the interior and exterior of a building wall, water vapor will transport through the wall from the side with higher humidity to the side with lower humidity; this phenomenon is widely used as a crucial mass transfer parameter in wall insulation research [1,2]. When the water vapor transmission property of the waterproof mortar on the outer layer of the building structure is poor, water vapor may accumulate at the interface due to hindrance. At this point, if there is a significant temperature difference between the indoor and outdoor environments, a large amount of water may condense, which is harmful to the service performance and life of the waterproof mortar [3]. Though the water vapor transmission property affects the service performance of waterproof mortar, current research on the factors and mechanisms influencing the water vapor permeability of mortar is relatively insufficient. The introduction of modifiers is a solution to this problem. There are various studies on modifiers for waterproof mortar [4,5,6,7], but there is limited discussion on how to make mortar have both excellent waterproofness and water vapor transmission property. The modifiers can be divided into three main categories: densifiers, water repellents, and crystalline admixtures [6]. Among them, cementitious capillary crystalline waterproof materials (CCCW), silicone-based compounds, and polymer modifiers are excellent and representative of the primary action of waterproofing modifiers. Therefore, this paper adopts these three representative waterproof modifiers to modify mortar, and studies its waterproofness, impermeability, and water vapor transmission property, along with conducting a mechanistic analysis.

A previous study [7] has shown that the diffusion process of water vapor in porous media is essentially the transport within the pore structure controlled by the humidity gradient pressure. Therefore, after conducting some surveys and preliminary experiments, this study selects three waterproof modifiers with different impacts, namely polymer modifier, silicone modifier, and cementitious capillary crystalline waterproof material modifier [4,5,7]. CCCWs such as XYPEX have a complex composition, mainly consisting of Portland cement, active chemical substances, and complex catalysts [5,8]. These materials can improve the mechanical strength of the modified mortar [9] and fill pores in the mortar through crystallization reactions, reducing its porosity [10], and impart self-healing capabilities to the mortar [10,11]. Silicone, such as γ-methacryloxy-propyl-trimethoxy-silane (KH570), primarily enhances waterproof performance by imparting hydrophobicity to the mortar [4,12,13], which can endow mortar with excellent waterproof performance according to references [12,13] and the preliminary experiment. Additionally, KH570 has air-entraining and water-reducing effects, which can increase the porosity of the modified mortar to a certain extent [14]. However, studies [12,14] have shown that silicones mostly reduce the mechanical properties of the modified mortar and delay the hydration. Polymer modifier imparts excellent waterproof properties to mortar by forming polymer film networks [15,16,17]. Additionally, polymers generally have an air-entraining effect [18], and improve the porosity of the modified mortar [19,20]. In this paper, styrene-butadiene rubber latex (SB) is selected as the polymer. It can improve various aspects of mortar performance [21,22,23], such as increasing the fluidity of cement paste, enhancing flexural strength and bond strength, and improving the durability of hardened paste. Previous experiments [24,25] show that SB can increase the air content of freshly mixed cement paste, reduce its bulk density, and optimize its pore structure.

Previous studies have not comprehensively evaluated the influencing factors of waterproof, impermeability, and water vapor transmission property, and most of them are studies of waterproof and impermeability, or only analyses of water vapor transmission [7,8,9,10,11,12]. As a result, the aims of this study are to investigate the effects of different modifiers on the waterproofness, impermeability, and water vapor transmission property of mortar, discuss their impact mechanisms, and explore modifiers that can simultaneously improve both water vapor transmission property and waterproofness. The waterproof performance of mortar is characterized by the capillary water absorption (CWA); the impermeability is characterized by the impermeability pressure; and the water vapor transmission property is characterized by the water vapor permeability by the gravimetric method [1]. Porosity and pore size distribution are significant factors affecting the waterproofness and water vapor transmission property of mortar, which are characterized by mercury intrusion porosimetry (MIP) in this study. Additionally, the micromorphology of the mortar is observed by scanning electron microscopy (SEM). The hydrophobicity of the mortar is characterized by the water contact angle. Low-field nuclear magnetic resonance (LF-NMR) is employed to study the water distribution when water vapor transmission reaches a steady state. The degree of hydration is characterized by X-ray diffraction (XRD). Furthermore, based on mechanistic analysis, the relationship between different modifiers and properties of mortar is established by pore properties. This study can optimize the performance of mortar’s properties of waterproof, impermeability and water vapor transmission, and provide a theoretical reference for the control of mortar’s waterproof, impermeability and water vapor transmission.

## 2. Materials and Methods

### 2.1. Materials

P·II 52.5R Portland cement (PC) is produced by Anhui Conch Cement Co., Ltd., Anhui, China. The main chemical composition is shown in Table 1.

Quartz sand is produced by Shanghai Fengchen Powder Material Co., Ltd., Shanghai, China. G3 (10–20 mesh), G4 (20–40 mesh), G6 (40–80 mesh) and G7 (80–120 mesh) are used, mixed by 35%, 30%, 25%, 10%. The fineness modulus is 2.55, and the remaining screening is shown in Table 2.

**Figure 1:**
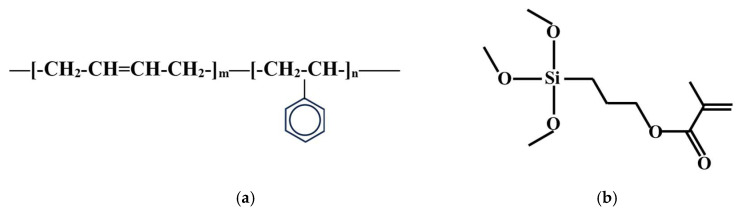
(**a**) The approximate chemical structure of SB; (**b**) The structural formula of KH570.

The polymer emulsion Styrofan^®^ ECO-7623 (SB), produced by BASF SE, Shanghai, China, has a solid content of 51% ± 1%, an average particle size of 160.6 nm, a viscosity of 35–150 mPa·s, and a minimum film forming temperature of 14 °C. The approximate chemical structure of SB is shown in Figure 1a.

XYPEX admixture is a kind of cementitious capillary crystalline waterproof material produced by XYPEX^®^, Hong Kong, China. The main chemical composition of XYPEX is shown in Table 3.

The γ-methacryloxy-propyl-trimethoxy-silane (KH570), produced by Changzhou Runxiang Chemical Co., Ltd., Changzhou, China., has the structural formula shown in Figure 1b. The density is 0.94–0.99 kg/L, and the active component content is ≥98%.

The water is Shanghai tap water, and the mass ratio of cement to sand was 1:3. The XYPEX content (relative to cement mass) is 0.5%, 1%, 1.5%, 2%, noted by X1, X2, X3, X4, respectively. The KH570 content is 1.25%, 2.5%, 3.75%, 5%, noted by K1, K2, K3, K4, respectively. The content of SB (based on solid content) is 5%, 10%, 15%, 20%, noted by S1, S2, S3, S4, respectively; the comparison sample is noted by C, with a total of 13 groups, as shown in Table 4. The content of the mixture is determined by the previous study and the manufacturer’s suggestion. According to standard [26], the water-to-cement ratio is changed to adjust the fluidity of mortar to (170 ± 5) mm. The water-to-cement ratio after adjustment is shown in Table 4. 

### 2.2. Preparation of Mortar and Paste 

The preparation of fresh mortar refers to the standard [27]. XYPEX and cement are dry-mixed evenly before the mortar is mixed. SB is mixed with water. KH570 is added drop by drop, 10 s after the mortar is mixed. The cement and sand are dry-mixed evenly in the mixer, and the preparation of mortar begins after adding water. The fresh mortar is obtained by mixing at a slow speed for 60 s, followed by a fast speed for 30 s, then stopping for 90 s, and finally mixing at a fast speed for 60 s. After the mortar mixing is completed, it is poured into the mold in two layers. After vibration to compact the mixture, the upper surface is leveled with a spatula. The mold is then covered with plastic wrap and cured in a curing chamber at a temperature of (20 ± 2) °C and a relative humidity of (60 ± 5)% for 24 h before demolding. Subsequently, the samples continue to be cured under the same conditions for 28 days.

The preparation of cement paste follows the standard [28]. Cement paste is only used in X-ray diffraction. The mixing procedure for the modified material is consistent with that of the mortar, involving slow mixing on a mixer for 120 s, a pause of 15 s, and then fast mixing for another 120 s to obtain the fresh paste. The well-mixed cement paste is then poured into 40 mm × 40 mm × 40 mm molds in two layers. After vibration to compact the paste and leveling the upper surface, the molds are covered with plastic wrap. The curing time and conditions are the same as those for the mortar.

### 2.3. Methods

#### 2.3.1. Capillary Water Absorption

Capillary water absorption is a representative factor to characterize waterproofing, which shows the ability of mortar to absorb water. It is a factor that describes the variation in water absorption per unit area of mortar with the time of cube opening. The test method for capillary water absorption follows the standard [29]. For each group, three samples with dimensions of 40 mm × 40 mm × 160 mm are molded. After 28 days of curing, the samples are dried in an oven at 40 °C for 72 h until they reach a constant weight. Then they are cooled to room temperature, and the surfaces of the samples are sealed with paraffin wax, except for the molding surface and its opposite surface, which remain unsealed. After sealing, the area of the molding surface is measured, and the mass of each sample is weighed by a balance with a precision of 0.01 g. The samples are then submerged with their upper surfaces facing downwards, 2–3 mm into the water, using a 6 mm diameter glass rod to support the sample above the bottom of the water container. When weighing the samples after water absorption, water droplets on the surface need to be wiped away using a wrung-out wet towel, and the change in sample mass over time is recorded.

#### 2.3.2. Impermeability

Impermeability pressure is a representative factor to characterize impermeability. It is a factor that describes the ability to resist osmotic pressure within a certain period of time. The molding and testing methods for impermeability pressure samples follow the standard [30]. Each group consists of six samples, molded using a frustum-conical mold with an upper diameter of 70 mm, a lower diameter of 80 mm, and a height of 30 mm. After curing for 28 days, the lateral surfaces of the mortar samples are sealed with paraffin and waterproof glue, and then the samples are fixed to the impermeability tester using screws. The initial impermeability pressure is set at 0.2 MPa, held constant for 2 h, and then increased to 0.3 MPa. Subsequently, the pressure is increased by 0.1 MPa per hour until it reaches 1.5 MPa. When water seepage is observed on the surfaces of three samples in a group, the test for this group is stopped, and the water pressure minus 0.1 MPa at this point is recorded as the impermeability pressure for this group. If no water seepage is observed after maintaining a pressure of 1.5 MPa for one hour, the test is stopped, and the samples are vertically split along their centerline to measure the depth of water penetration.

#### 2.3.3. Water Vapor Transmission Property

**Figure 2:**
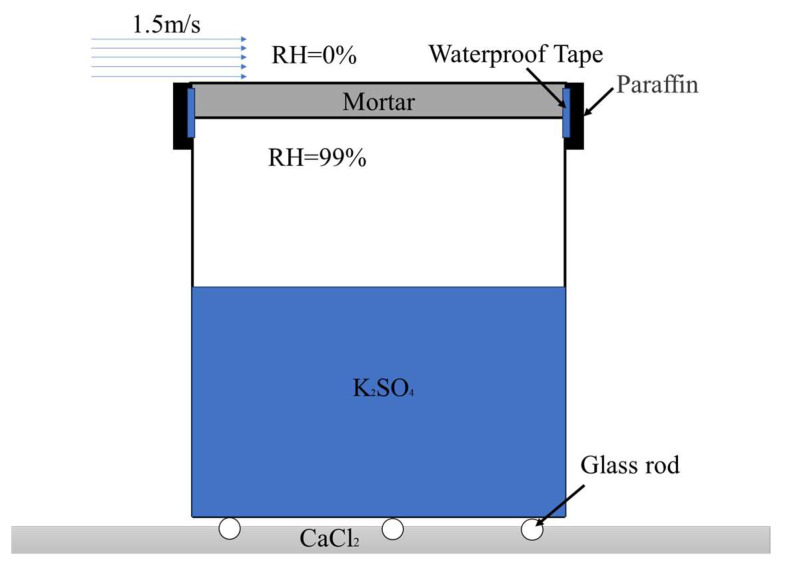
Schematic diagram of the test cup for water vapor transmission property.

The water vapor transmission property is characterized by the water vapor permeability, an intensive property that measures the inherent ability of a material to allow water vapor transmission, calculated based on the density of water vapor flow rate. The testing method for the density of water vapor flow rate employs the gravimetric method, referring to [31]. The mortar is molded into 142 mm diameter and 13 mm high circular disc-shaped samples, with a curing period of 28 days. The test cups have an outer diameter of 140 mm and a height of 150 mm. The laboratory temperature is maintained at (20 ± 1) °C, with the interior of the test cups filled with supersaturated potassium sulfate solution (K_2_SO_4_) to control the relative humidity at 99%. The exterior is dried using anhydrous calcium chloride (CaCl_2_), and a hygrometer is used to monitor the relative humidity on the upper surface of the samples. A fan provides a wind speed of (1.5 ± 0.2) m/s on the upper surface. Prior to testing, the samples are cured in a dry room at a temperature of (20 ± 1) °C and a relative humidity of (60 ± 5)%. At the start of the test, the test cups are assembled as shown in Figure 2, with the sides fixed by waterproof tape and sealed with paraffin. The test cups are then placed inside a sealed box filled with anhydrous calcium chloride, and the mass of the test cups is measured every 12 h using a balance with an accuracy of 0.01 g. When the fluctuation range of the mass change rate is within ±5% for five consecutive measurements, the test cups are considered to have reached a steady state, and the density of water vapor flow rate and water vapor permeability of the specimens are calculated using Equations (1) and (2).

(1) $$\delta =d\frac{g}{∆p}=d\frac{g}{p_{s}(R_{H1}-R_{H2})}$$

δ—Water vapor permeability, kg/(m∙s∙Pa);

g—Density of water vapor flow rate, kg /(m^2^∙ s);

d—Thickness of samples, m;

∆p—Pressure difference between the two sides of the samples, Pa;

$p_{s}$—Saturated vapor pressure at the test temperature, Pa;

$R_{H1}$—Relative humidity on the high humidity side, %;

$R_{H2}$—Relative humidity on the low humidity side, %.

(2) $$g=\frac{∆m_{t}}{∆t}/A$$

g— Density of water vapor flow rate, kg /(m^2^·s);

$∆m_{t}$—Mass change of the test cup in time *t*, kg;

∆t—Time *t*, s;

A—Effective area of samples, m^2^.

#### 2.3.4. Mercury Intrusion Porosimetry

The porosity and pore size distribution of mortar after 28 days of hardening are tested by mercury intrusion porosimetry (MIP). To prepare the MIP samples, the samples are crushed into particles with a diameter of 5–7 mm, soaked in anhydrous ethanol, with the anhydrous ethanol replaced every 2 days for a soaking duration of 7 days to ensure complete termination of the hydration reaction. Subsequently, the small samples are placed in a vacuum oven at 40 °C and dried until they reach constant weight, followed by sealing in a vacuum bag for testing. The porosity and pore size distribution of the hardened cement paste are measured by a Micromeritics Autopore V 9620 MIP instrument from the Norcross, GA, USA, with a pore size measurement range of 5 nm to 800 μm.

#### 2.3.5. Water Contact Angle

**Figure 3:**
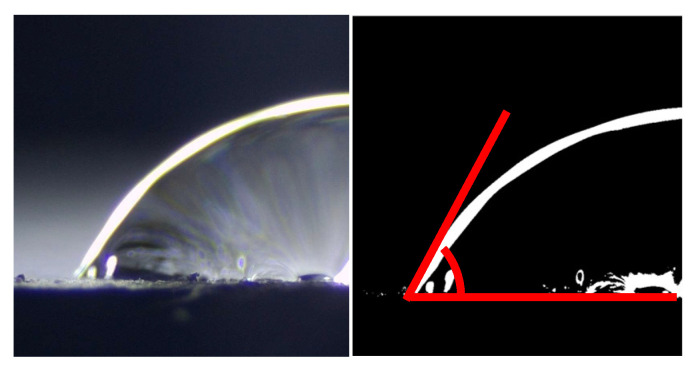
Untreated (**left**) and binarized (**right**) photos of the water contact angle test.

The hydrophobicity of mortar is characterized by measuring its water contact angle. To more accurately represent the hydrophobicity within the mortar, freshly cut sections are selected for testing. After curing the mortar samples for 28 days, they are sectioned using a cutting machine. Subsequently, a 5 μL droplet of deionized water is placed 3 mm above the height of the fresh section. A photograph is taken 1 s later and binarized for analysis, as shown in Figure 3. The final contact angle value for each sample is obtained by averaging the results of six tests.

#### 2.3.6. Scanning Electron Microscopy

Microscopic morphology of mortar hardened for 28 days is observed by scanning electron microscopy (SEM). To prepare the SEM samples, the samples are first crushed into pieces with a width of approximately 8 mm and a thickness of approximately 3 mm, where the observation surface is a fresh fracture that is as flat as possible. After terminating the hydration reaction, the samples are dried to a constant weight in a vacuum oven. Samples are then fixed onto the sample stages using conductive adhesive and coated with gold. Surface morphology is observed using a ZEISS Sigma 300VP field emission scanning electron microscope from Oberkochen, Germany.

#### 2.3.7. Low-Field Nuclear Magnetic Resonance

The internal pore water distribution of mortar after water vapor transmission reaches a steady state is characterized using low-field nuclear magnetic resonance (LF-NMR). Firstly, the samples with stabilized mass changes from the water vapor transmission property test are cut into rectangular prism samples with dimensions of 15 mm × 10 mm × 80 mm and immediately placed into NMR tubes for transverse relaxation testing to obtain the T_2_ spectrum through inversion. After the test, the sample mass is recorded using an analytical balance, and the samples are then placed in a vacuum oven at 40 °C. Once the samples are dried to a constant mass, their mass is recorded again to calculate the mass loss during the drying process. The dried samples are then placed back into NMR tubes for additional testing. The low-field NMR instrument used is the MicroMR12-025V from Suzhou, China, operating at 12 MHz, employing the Carr-Purcell-Meiboom-Gill sequence for testing, magnetic field strength: 0.49 T, echo time: 100 μs, scan times: echoes: 4000, signal-to-noise ratio: >100, the inversion method: SIRT, iterations: 10,000.

#### 2.3.8. X-Ray Diffraction

The impact of various modifiers on cement hydration is analyzed by X-ray diffraction (XRD). The cement paste samples cured for 28 days are crushed. After the termination of the hydration reaction, the samples are subsequently placed in a vacuum oven at 40 °C until they reach a constant weight. The crushed sample fragments are then ground into a powder using an agate mortar and passed through a 250-mesh sieve. The sieved powder samples are placed in a desiccator for testing. Testing is conducted using a Rigaku Smart Lab SE X-ray powder polycrystalline diffractometer from Japan with a copper target as the X-ray generator, a 2*θ* test range of 5° to 70°, a scan speed of 2°/min, and a step size of 0.01°.

## 3. Results and Discussion

### 3.1. Capillary Water Absorption

**Figure 4:**
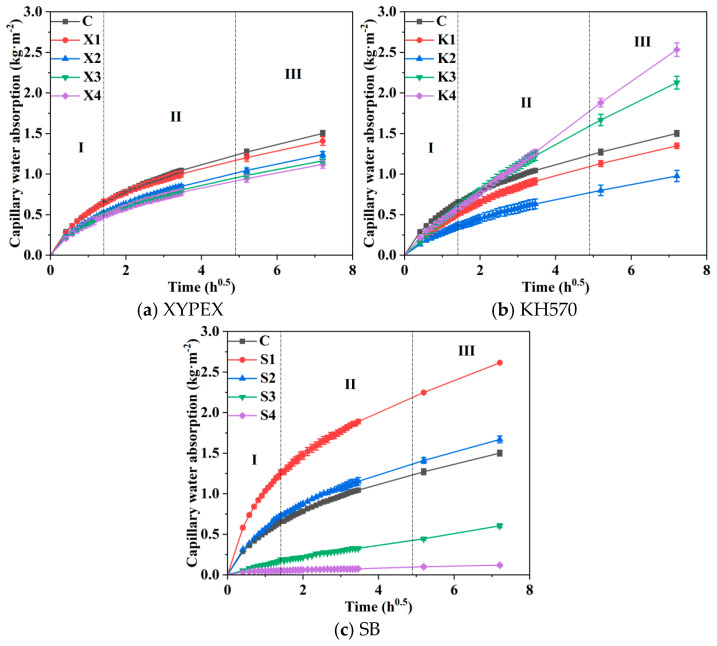
Capillary water absorption of different modified mortars: (**a**) XYPEX, (**b**) KH570, (**c**) SB.

Figure 4 presents the CWA of different modified mortars. The trend of performance changes is primarily analyzed in this section, and further mechanistic analysis requires consideration of porosity and pore characteristics, which are discussed later. Overall, the CWA of all modified mortars gradually increases with immersion time. Based on reference [32], the water absorption curves of the modified mortars are divided into three stages: rapid water absorption stage (I, 0 h to 2 h), slow water absorption stage (II, 2 h to 24 h), and stable water absorption stage (III, 24 h to 48 h). Linear fitting is performed on the water absorption curves of each stage to calculate the capillary water absorption coefficient, which represents the water absorption rate for that stage. The results are shown in Table 5. 

The experiments indicate that a higher content of XYPEX in the modified mortar leads to a lower CWA. When the content of XYPEX is 0.5%, its CWA is 6% lower than that of the comparison sample, with a CWA of 1.41 kg·m^−2^ at 48 h. When the content is 1% or above, the CWA of the modified mortar decreases significantly. As the content increases, the CWA further decreases, though not to a significant extent. Among them, the CWA at a content of 2% is the lowest, with a CWA of 1.12 kg·m^−2^ at 48 h, which is 25.3% lower than the comparison sample. In addition, XYPEX significantly reduces the capillary water absorption coefficient at each stage.

At low contents, KH570 reduces the CWA of modified mortar, but when the content of KH570 is too high, the CWA of the modified mortar increases compared to the comparison sample. At contents of 1.25% and 2.5%, KH570 exhibits lower CWA of 1.35 kg·m^−2^ and 0.98 kg·m^−2^, respectively, which are 10.3% and 35% lower than the comparison sample. When the content of KH570 is increased to the higher levels of 3.75% and 5%, the higher the content, the higher the CWA. Specifically, at a content of 5%, the CWA is 2.53 kg·m^−2^, which is 68.7% higher than that of the blank group. At low contents, KH570 also reduces the capillary water absorption coefficient at each stage, with the most significant reduction occurring in the early stages. At a content of 2%, the coefficient is 0.26 kg·m^−2^·h ^−1/2^, which is 44.7% lower than the comparison sample.

At high contents of SB, the CWA decreases significantly. However, the CWA of the modified mortar generally increases at a lower content. When the SB content is 5%, the CWA at 48 h is 2.61 kg·m^−2^, 74.0% higher than that of the comparison sample. As the SB content increases to 10%, the CWA decreases compared to the 5% content, but remains slightly higher than the comparison sample. When the content reaches 15%, the CWA of the modified mortar decreases greatly, with a CWA of 0.61 kg·m^−2^ at 48 h, 59.3% lower than the comparison sample. When the SB content is 20%, the CWA further decreases to 0.12 kg·m^−2^, 92.0% lower than the comparison sample. At a content of 15%, SB significantly reduces the early-stage capillary water absorption coefficient. Specifically, the coefficient in stage I is 0.12 kg·m^−2^·h^−1/2^, 74.5% lower than the comparison sample. When the content is 20%, the capillary water absorption coefficients in stages II and III are 0.01 kg·m^−2^·h^−1/2^, indicating that capillary water absorption almost ceases at this point.

After comprehensively comparing the three modifiers, it is found that 20% SB exhibits the most significant effect in reducing CWA, while SB at contents of 5% and 10% increases the CWA. XYPEX can significantly reduce the CWA, and the higher the content, the more pronounced the reduction effect. KH570 performs excellently at contents of 1.25% and 2.50% but poorly at contents of 3.75% and 5.00%. Based on the performance in reducing CWA, the ranking is S4 > S3 > K2 > X4. Low SB content and high KH570 content are detrimental to reducing the CWA of mortar.

### 3.2. Impermeability

**Figure 5:**
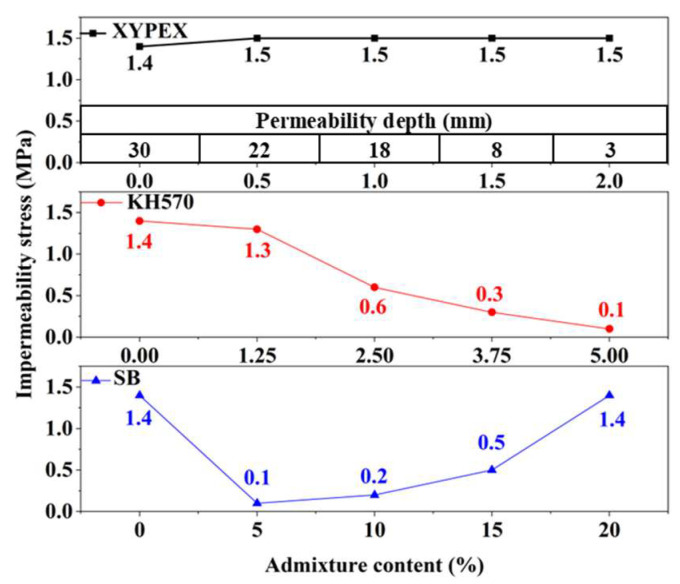
The impermeability pressure and permeability depth of different modified mortars.

The results of the impermeability tests for different modified mortars are shown in Figure 5. The impermeability pressure of mortars modified with KH570 and SB is less than 1.5 MPa, and the impermeability pressure is used to characterize their impermeability performance. The XYPEX-modified mortar does not leak under a water pressure of 1.5 MPa, so the permeability depth is used as a supplementary characterization of its impermeability performance. The experiments indicate that modifying mortar with XYPEX can significantly enhance its impermeability, and this enhancement increases with the content of XYPEX. At a content of 0.5%, the impermeability pressure of XYPEX can reach above 1.5 MPa, and at a content of 2%, the penetration depth is only 3 mm under a water pressure of 1.5 MPa, showing its excellent impermeability performance. As the content of KH570 increases, the impermeability of the modified mortar decreases significantly. At a content of 1.25%, the impermeability pressure of the mortar slightly decreases to 1.3 MPa, and at a content of 5%, the modified mortar almost loses impermeability. The impermeability performance of SB-modified mortar is extremely poor at low contents, but its impermeability improves as the content increases. At a content of 20%, its impermeability is basically the same as that of the comparison sample.

### 3.3. Water Vapor Transmission Property

**Figure 6:**
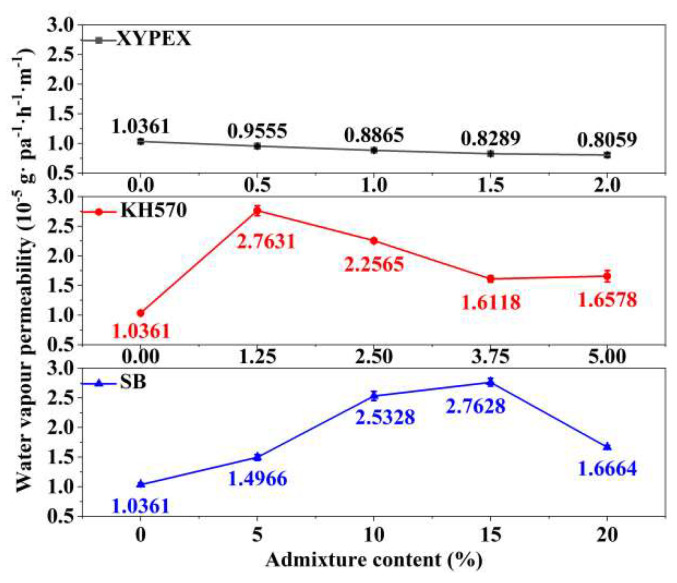
Water vapor permeability of different modified mortars.

The results of the water vapor transmission property tests for modified mortars are shown in Figure 6, characterized by the water vapor permeability. A higher water vapor permeability indicates better water vapor transmission property of the material, which represents a better passage of water vapor. Compared to the comparison sample, XYPEX-modified mortar exhibits a lower water vapor permeability, and as the content increases, the water vapor transmission property gets worse. Specifically, at a content of 2.0%, the water vapor permeability of XYPEX-modified mortar is 22.2% lower than that of the comparison sample. Overall, KH570 can improve the water vapor transmission property of mortar, but as the content increases, these properties gradually decline. When using a 1.25% content of KH570 to modify mortar, its water vapor permeability increases by 166.7% compared to the comparison sample; however, at a content of 5%, the increase is only 60.0%. SB can enhance the water vapor transmission property of mortar. At contents below 15%, the higher the content, the higher the water vapor permeability of the modified mortar. At a content of 15%, there is a 166.6% increase, but at a content of 20%, the water vapor permeability of the modified mortar only increases by 22.0% compared to the comparison sample.

### 3.4. Mechanism Analysis

Based on the performance tests conducted in Section 3.1, Section 3.2 and Section 3.3, it is evident that XYPEX can significantly enhance the impermeability of mortar and improve its waterproofness to a certain extent, but it reduces the water vapor transmission of mortar, with the effect more pronounced at higher content levels. At low content levels (1.25% and 2.5%), KH570 can enhance the waterproofness of mortar and markedly improve its water vapor transmission property; however, it also slightly decreases the impermeability. At high content levels, KH570 exhibits poor performance in terms of waterproofness, impermeability, and water vapor transmission property. When SB is used at high content levels (15% and 20%), it shows exceptional waterproofness and water vapor transmission properties to the mortar but results in poorer impermeability. Notably, at an SB content of 20%, the waterproofness is excellent, accompanied by some improvement in water vapor transmission property and no significant decrease in impermeability. However, at lower content levels, SB’s related performances are less satisfactory. To analyze the mechanism of action, eight groups of samples, C, X2, X4, K2, K4, S2, S3, and S4, are selected to characterize their properties by MIP, water contact angle test, SEM, LF-NMR, and XRD.

#### 3.4.1. Porosity and Pore Size Distribution

**Figure 7:**
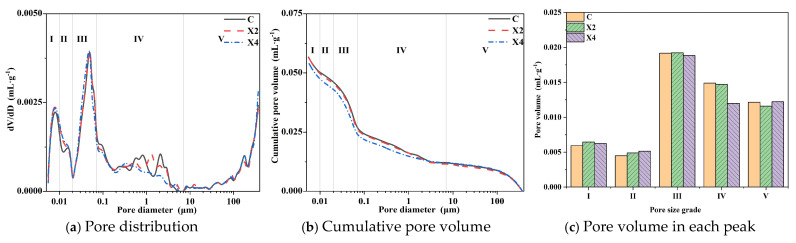
The influence of XYPEX on the pores of mortars.

**Figure 8:**
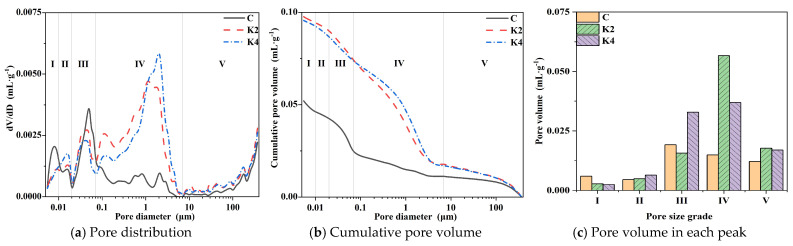
The influence of KH570 on the pore of mortars.

**Figure 9:**
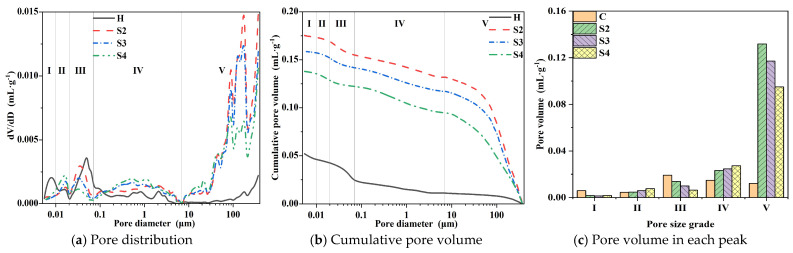
The influence of SB on the pores of mortars.

The waterproofness, impermeability, and water vapor transmission property of mortar are closely related to its internal pores. In order to analyze the effect of modified mortar pores, the pore structure of the hardened mortar is tested by MIP. According to the pore size, the internal pores of mortar can be generally categorized into gel pores (<10 nm), transition pores (10–100 nm), capillary pores (100 nm–1 μm), and macropores (>1 μm) [32]. Figure 7, Figure 8 and Figure 9 show the cumulative pore volume and pore size distribution of the modified mortars. It can be seen that there are mainly five peaks of <10 nm, 10–20 nm, 20–70 nm, 70 nm–7 μm, and >7 μm, which are considered as gel pores (I), transition pores (II), small capillary pores (III), large capillary pores (IV), and air pores (V), respectively. The results of average pore diameter, median pore diameter, mode pore diameter, and porosity tested by MIP are shown in Table 6.

According to Figure 7, the results show that XYPEX reduces the porosity of the modified mortar. When the XYPEX content is 2%, the porosity is 12.0%, 3.3% lower than that of the comparison sample. Though it shows that peaks II and IV have obvious changes, when the XYPEX content is 2%, the changes in porosity between the comparison mortar and XYPEX-modified mortar are small. So, the conclusion we can draw is that XYPEX could decrease large capillary pores to some degree, and the change in porosity is inapparent. XYPEX is a kind of CCCW with a fine particle size and active ingredients. It promotes hydration through complexation-precipitation reactions, generating more hydration products in the pores [11], blocking the pores within the mortar, reducing the number of capillary pores and air pores, and increasing the compactness of the modified mortar. Therefore, after modifying the mortar with XYPEX, the porosity of the mortar decreases, but inapparent, capillary pores decrease, and its capillary action weakens. The mortar becomes more compact, leading to a reduction in CWA, enhanced impermeability, and decreased water vapor transmission property.

KH570 increases the porosity of the modified mortar. When the content is 2.5% and 5%, it increases by approximately 90% compared to the comparison sample. KH570 mainly introduces large capillary pores, with a slight increase in air pores but a significant reduction in gel pores and small capillary pores. The average pore diameter, median pore diameter, and mode pore diameter of the modified mortar are all significantly increased. At a content of 2.5%, the mode pore diameter increases from 50.4 nm in the comparison sample to 2.5 μm. Tests show that KH570 introduces a large number of capillary pores and air pores, significantly reducing the compactness of the mortar. The results draw that KH570 mainly introduces large capillary pores. According to reference [33,34], KH570 partly hydrolyzes into silanol and methanol in the alkaline mortar pore solution, which changes the surface tension of the mortar and enables the bubbles to exist more stably in the mortar. So the amount of large capillary pores is included as the results show. During impermeability tests, water has more capillary pores and air pores to invade under the action of water pressure, explaining the decrease in the impermeability of the mortar. In addition, the increase in capillary pores should theoretically enhance the CWA of the mortar. However, experiments indicate that the CWA of the mortar with a 2.5% KH570 content is significantly reduced compared to the comparison sample. This is because the KH570-modified mortar exhibits hydrophobicity. Upon contact with water, it hydrolyzes into silanol and methanol. Silanol can undergo dehydration condensation by itself or with c on the surface of hydration products, forming a thin film of silanol condensation on the surface. Due to the hydrophobicity of the organic groups at the other end, the film formed inside the mortar is hydrophobic [34]. Therefore, although the porosity of the mortar increases significantly at a 2.5% KH570 content, the hydrophobicity imparted by KH570 reduces the capillary action of the capillary pores. Consequently, despite the introduction of more capillary pores and air pores, the CWA of the mortar decreases at a 2.5% content. In contrast, the increase in CWA at a 5% content may be attributed to self-dehydration condensation dominating at high KH570 contents, which significantly increases steric hindrance during hydration and reduces condensation with the surfaces of hydration products, ultimately resulting in poorer hydrophobic modification of the mortar. Therefore, in Section 3.4.2, the hydrophobicity of the modified mortar will be characterized to further analyze the mechanism of action.

SB significantly increases the porosity of the modified mortar, with S2, S3, and S4 increasing by 133.0%, 120.5%, and 99.4% compared to the comparison sample. The increase in porosity of SB-modified mortar is due to the substantial introduction of air pores. Compared to KH570, which mainly introduces large capillary pores, SB primarily introduces air pores. Additionally, compared to C, the capillary pores in SB-modified mortar decrease, with a significant reduction in gel pores. The average pore diameter, median pore diameter, and mode pore diameter of the modified mortar all increase significantly, with the mode pore diameter of S2 increasing from 50.4 nm to 181.1 μm. As the content increases, the average pore diameter, median pore diameter, and mode pore diameter decrease in some degree, with the mode pore diameter of S4 decreasing to 90.5 μm. This is because SB has a significant air-entraining effect, which reduces the interfacial tension between water and air in the paste, stabilizes the bubbles introduced during mixing, and enables smaller pores to fuse into larger, stably existing pores [23]. Furthermore, SB can form a polymer film in the mortar [15,16], sealing some of the smaller gel pores and capillary pores. Therefore, at higher contents, although SB introduces a large number of air pores, it also forms more continuous polymer films inside, sealing the capillary pores and gel pores. Simultaneously, the large number of air pores disrupts the continuity of the capillary pores in the mortar, cutting capillary water absorption and enhancing the waterproofness of the mortar. At lower SB contents, the air-entraining effect is pronounced, resulting in a higher porosity and the introduction of a large number of air pores and capillary pores. At the same time, due to the poor sealing of the polymer film and the excellent connectivity between air pores, the impermeability of the modified mortar decreases significantly, and the capillary water absorption rate is higher than that of the comparison sample. However, at higher contents, the polymer film forms more completely, with better sealing and poorer connectivity between air pores, leading to a significant decrease in capillary water absorption rate and an increase in impermeability.

#### 3.4.2. Water Contact Angles

**Figure 10:**
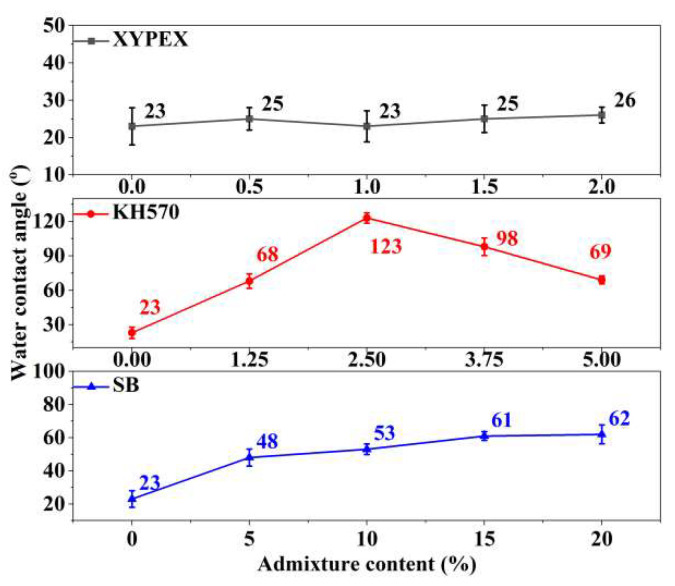
Water contact angles at profiles of different modified mortars.

Figure 10 presents the water contact angles of different modified mortars. The addition of XYPEX has no significant effect on the water contact angle of the mortar surface. This is because its composition is primarily inorganic, consisting mostly of active substances similar to cement components. Its mechanism of action involves promoting hydration via complexation-precipitation reactions, generating hydration products, and blocking pores, with little impact on the surface free energy of the mortar [11]. Therefore, the water contact angle of the mortar remains consistent with that of the comparison sample.

Admixing an appropriate content of KH570 significantly improves the hydrophobicity of the mortar. When a small content (1.25%) of KH570 is added, the water contact angle increases to 68°, showing a notable improvement. At a 2.5% KH570 content, the contact angle reaches 123°, indicating excellent hydrophobicity of the mortar. However, as the content further increases, the water contact angle decreases, dropping to 69° at a 5% content. The hydroxyl groups of silanol formed after the hydrolysis of KH570 can undergo dehydration condensation with the hydroxyl groups in the mortar, coating its surface with a layer of hydrophobic functional groups. This reduces its surface tension and the contact angle with water, thereby achieving a hydrophobic effect [6,14]. However, when the content of KH570 is too high, the hydrophobic modification effect deteriorates, and the mortar loses its hydrophobicity. When the KH570 is at high concentration, KH570 and the silanol produced by hydrolysis will cause agglomeration [12], resulting in uneven dispersion and the waterproof film forming badly. That is the reason why the mortar loses its hydrophobicity at high content of KH570. Meanwhile, as indicated in Section 3.4.1, the mortar exhibits an obvious air-entraining effect at this point, with a mode pore diameter of 1.05 μm. The pores introduced are mostly large capillary pores, which can enhance the capillary action of the mortar. At lower KH570 contents, although the modified mortar introduces more capillary pores, its excellent hydrophobicity significantly weakens the capillary action of the pores, resulting in a substantial reduction in capillary water absorption (CWA) compared to the comparison sample. At higher contents, the modified mortar lacks hydrophobicity, leading to an increase in CWA compared to the comparison sample.

The addition of SB results in a certain increase in the water contact angle of the mortar, and as the content increases, the contact angle also increases slightly. At a content of 20%, the surface hydrophobic effect is the best, with a water contact angle of 63°, which still exhibits hydrophilic properties. According to reference [16], SB forms a polymer film inside the mortar, thereby reducing the surface tension of the mortar to a certain extent and increasing the contact angle. However, the increase in the water contact angle due to SB is relatively small, and it does not render the modified mortar hydrophobic.

#### 3.4.3. Micro-Morphology

**Figure 11:**
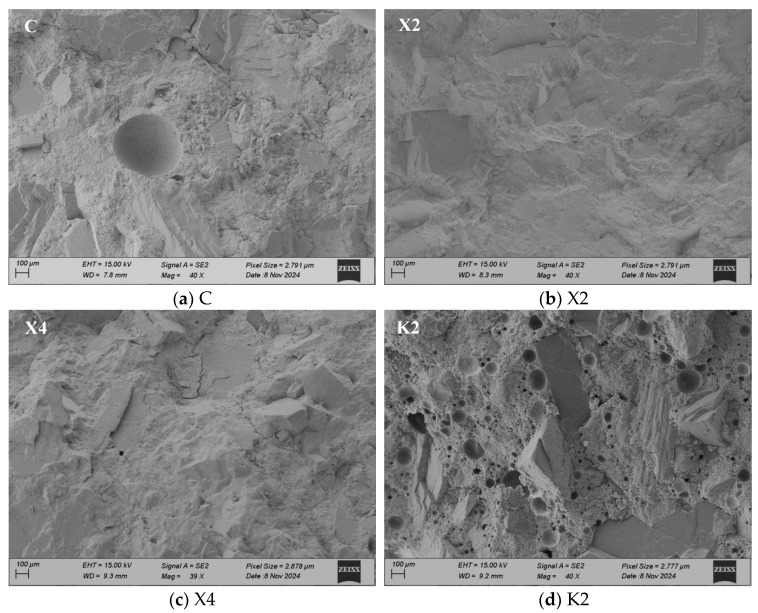

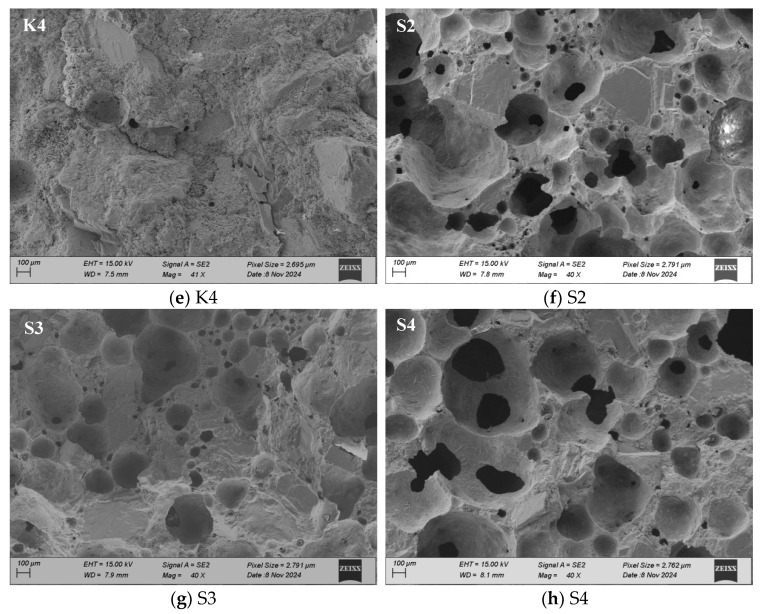
Air pores of different modified mortars under 40 times.

Figure 11 presents the air pore distribution of different modified mortars observed under an SEM at a magnification of 40 times. It can be observed in the figure that the XYPEX-modified mortar exhibits a similar characteristic to the comparison sample, with relatively few pores visible. The KH570-modified mortar, on the other hand, displays a relatively larger number of larger air pores and appears more porous compared to the comparison sample. Combined with MIP tests, KH570 contains a significant amount of large capillary pores in the micrometer range. For the SB-modified mortar, a large number of pores with sizes in the order of 100 micrometers can be clearly observed, and these pores are relatively regular and uniform in shape, presented as spheres. Based on the pore size distribution measured by MIP, XYPEX does not show air-entraining properties; KH570 introduces a large amount of large capillary pores ranging from 0.2 to 5 micrometers; and SB primarily introduces pores of 30 micrometers and above.

For SB-modified mortar, it can be observed in Figure 11 that the air pores introduced at a 20% content exhibit a relatively regular spherical shape. In contrast, although there are also numerous air pores present at a 10% content, there are more irregular pores larger than 200 nm. Comparing the pore size distribution diagrams of S2 and S4, it is evident that the air pores are divided into two peaks with 100 nm as the boundary, and S4 has significantly fewer pores larger than 100 nm compared to S2. At a 10% content, the air pores introduced by SB are numerous but not enough to form stable and regular bubbles. Additionally, the polymer film at this content has a weaker sealing effect on the capillary pores, resulting in better pore connectivity. Therefore, it provides numerous paths for water transport, leading to a substantial reduction in impermeability and an increase in CWA. When the SB content reaches 20%, the introduced air pores are more stable and independent, and the polymer film exhibits better sealing of the capillary pores at this point, resulting in poorer pore connectivity. This reduces the CWA of the mortar and restores its impermeability to a level consistent with the comparison sample, while the increase in water vapor transmission property becomes smaller. In summary, lower pore connectivity contributes to improving the waterproofness and impermeability of mortar but is detrimental to the water vapor transmission property.

**Figure 12:**
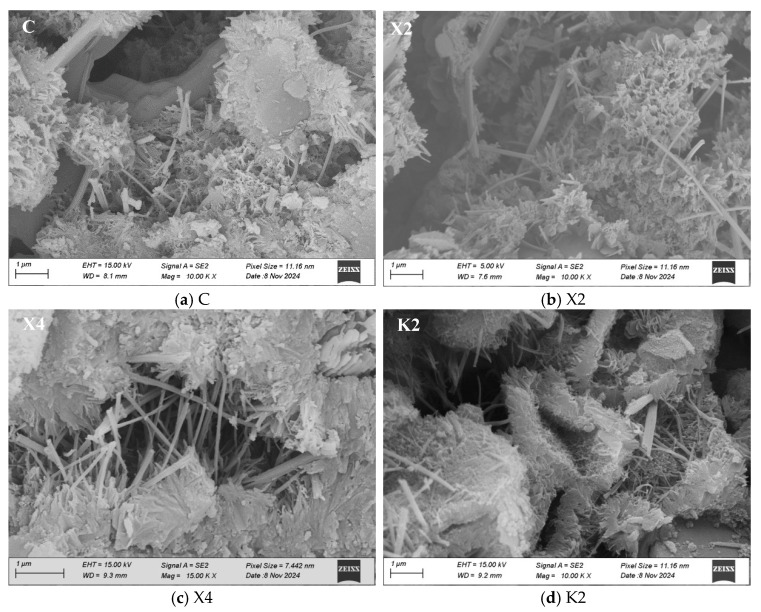

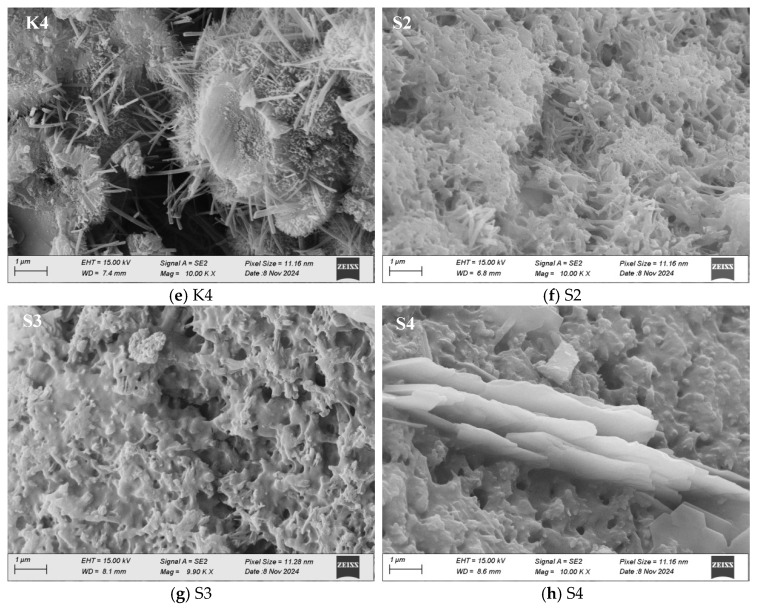
The microscopic morphology of different modified mortars.

Figure 12 presents the micro-morphology of different modified mortars observed under an SEM at a magnification of approximately 10,000 times. Compared to the comparison sample, XYPEX-modified mortar exhibits more hydration products within the pores, which supports the hypothesis that XYPEX can enhance the hydration of mortar, blocking pores and reducing porosity. It shows that the reduced porosity increases the compactness of mortar by generating hydration products, enabling it to resist water intrusion at higher pressures and exhibiting excellent waterproofness and impermeability. At the same time, the way for water vapor to transport is limited, decreasing the water vapor transmission. However, due to the high randomness of SEM observations, the degree of hydration will be assessed through XRD testing in Section 3.4.4 to validate this hypothesis. The hydration products in KH570-modified mortar exhibit poor connectivity, with numerous micrometer-sized pores present between the cement grains. The methanol and silanol hydrolyzed from KH570 alter the surface tension of the fresh mortar [4], thereby introducing more micrometer-sized pores. Additionally, the hydration products in KH570-modified mortar are relatively scattered, with the hydration process of cement delayed. This conclusion will be verified in Section 3.4.4. According to the micro-morphology result of KH570, when the content of KH570 is high, the hydration products are scattered and independent. It indicates that the KH570 at a high content will generates excessive silanol and methanol, which greatly improves the steric hindrance between hydration products. Due to the steric hindrance, the abundance of silanol may react with itself at a large amount, causing agglomeration, worsening the modification effect. In SB-modified mortar, a distinct polymer film can be observed, with cracks significantly reduced in the mortar. At high contents, a continuous polymer film encapsulating the hydration products can be seen, and it is difficult to find obvious cracks. This confirms that SB can form a polymer film on the mortar surface, capable of sealing small-sized pores. Therefore, the number of gel pores in SB-modified mortar is significantly reduced compared to the comparison sample.

#### 3.4.4. LF-NMR

**Figure 13:**
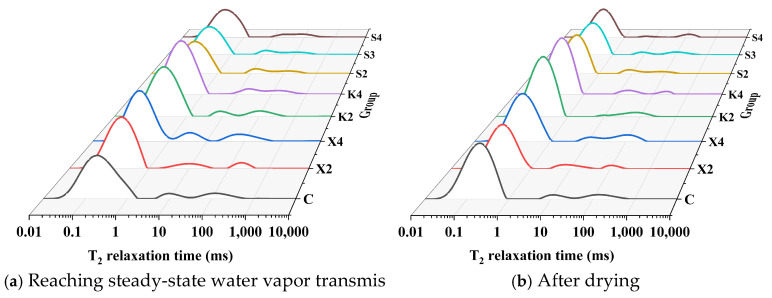
T_2_ spectra of different modified mortars.

Figure 13 presents the T_2_ spectra of different modified mortars after reaching a steady-state water vapor transmission level and after drying. Study [35] indicates a positive correlation between the volume of water and the LF-NMR signal intensity, with a correlation coefficient greater than 0.99. Relaxation time is related to pore diameter; thus, the type of water can be determined based on the peak’s position. Peaks located at less than 0.1 ms, 0.1 ms to 1 ms, 1 ms to 100 ms, and greater than 100 ms correspond to interlayer water, gel pores water, capillary water, and free water, respectively. In hardened mortar, interlayer water and gel water peaks often coalesce into one peak. Therefore, the three peaks in Figure 13, from left to right, can represent the content of interlayer and gel pores water, capillary water, and free water, respectively. According to Figure 13, after reaching a steady-state water vapor transmission level and after drying, the primary forms of water in the mortar samples are interlayer and gel pores. It can be observed that a certain amount of capillary water and free water still exists in the dried mortar samples. This is because the mortar contains a certain amount of closed pores that do not participate in water transport within the mortar. Therefore, by subtracting the peak areas after drying from those after reaching steady-state water vapor transmission, the distribution state of water in the pores after reaching steady-state water vapor transmission can be characterized. After the calculation, the proportions of water in various states for different modified samples are presented in Table 7.

**Figure 14:**
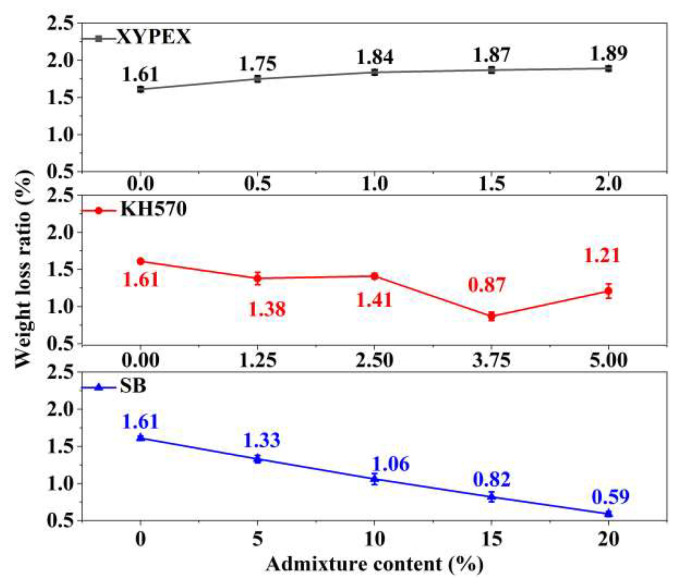
The weight loss ratio of different modified mortars after drying.

Figure 14 presents the weight loss rates of different modified mortars after drying, which represent the water adsorbed by the mortars after reaching steady-state water vapor transmission. Combining it with the water vapor permeability of different modified mortars shown in Figure 6, it can be observed that the lower the water vapor transmission of the modified mortar, the more water it adsorbs. According to Table 7, the adsorbed water vapor is primarily gel pores water, followed by capillary water. Combining this with the pore size distribution discussed previously, it can be seen that XYPEX-modified mortar exhibits a reduction in both capillary pores and air pores, with gel pores remaining basically unchanged; KH570-modified mortar reduces gel pores and mainly introduces larger capillary pores; and SB-modified mortar significantly reduces gel pores and mainly introduces air pores. Therefore, the porosity of large capillary pores in XYPEX-modified mortar decreases, making water vapor transmission more reliant on smaller gel pores and small capillary pores. A higher proportion of water vapor transmission is achieved through capillary condensation-evaporation, resulting in a higher mass of adsorbed water compared to the comparison sample. In contrast, KH570- and SB-modified mortars contain a large number of pores at the micrometer scale and above, with fewer gel pores capable of producing capillary condensation. Compared to the comparison sample, more water vapor passes through in the gaseous state, and less water undergoes capillary condensation within the pores. Additionally, water contact angle tests reveal that SB- and KH570-modified mortars exhibit higher hydrophobicity than the comparison sample. Therefore, under the same humidity, the critical diameter for capillary condensation is smaller, making it more difficult for capillary condensation to occur.

#### 3.4.5. Hydration Product

**Figure 15:**
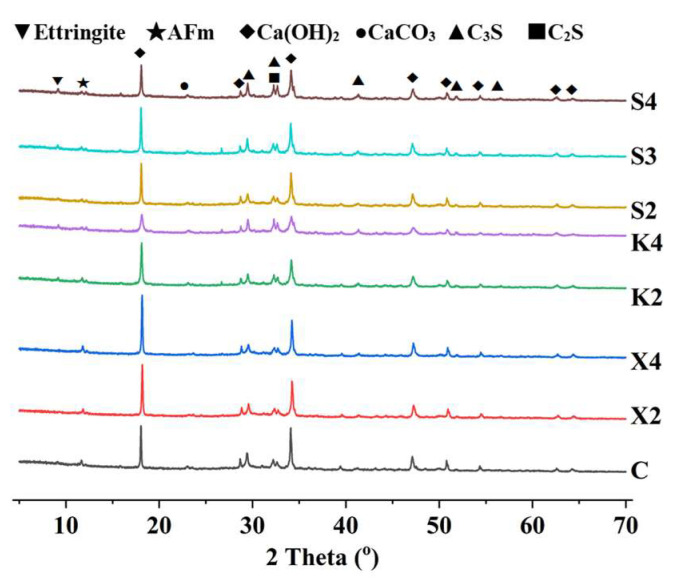
The XRD patterns of different modified cement pastes.

Figure 15 presents the XRD patterns of different modified cement pastes, with the main crystals being C_2_S, C_3_S, CaCO_3_, Ca(OH)_2_, AFm and ettringite [36]. The results indicate that XYPEX can significantly increase the content of Ca(OH)_2_ in the cement paste, simultaneously reducing the contents of C_3_S and C_2_S, with this reduction being more pronounced at higher contents. Therefore, XYPEX enhances the degree of cement hydration at 28 days. XYPEX is a kind of CCCW with fine particle size and active ingredients. It can carry out complexation-precipitation reactions [11]. The complex ions will combine with calcium ions in the pore solution and bring calcium ions to combine with other anion form precipitate. As a result, XYPEX can promote hydration through complexation-precipitation reactions, generating more hydration products in the pores, blocking the pores within the mortar, reducing the number of capillary pores and air pores and increasing the compactness of the modified mortar. The XRD result supports the hypothesis that XYPEX promotes cement hydration through complexation and precipitation reactions, explaining the observation in SEM of more hydration products in the pores of XYPEX-modified mortar. In contrast, the KH570-modified paste exhibits a lower content of Ca(OH)_2_ and relatively higher contents of C_3_S and C_2_S. Specifically, K2 slightly reduces the degree of hydration of the modified paste at 28 days, while K4 significantly reduces it. This suggests that excessively high KH570 content greatly increases the steric hindrance to cement hydration, hindering the hydration process. KH570 will hydrolyze into silanol and methanol in the alkaline environment of mortar. Silanol can condense and react with the hydroxyl groups in the C-S-H gel, forming a hydrophobic waterproof layer on the surface [14]. Therefore, it can increase the water contact angle of the mortar and make it hydrophobic. However, due to the increased steric hindrance of hydrolyzed silanol and methanol, the silanol reacted with hydration products that hinders the further development of hydration products and results in the presentation of separated granularity between hydration products. The contents of Ca(OH)_2_, C_3_S, and C_2_S in S2 and S3 are basically consistent with those in the comparison sample, showing no significant change in the degree of hydration of the paste at 28 days. However, the Ca(OH)_2_ content in S4 is lower than that in the comparison sample, indicating that styrene-butadiene latex modifier has a minor impact on the degree of hydration of the paste at 28 days when the content is 15% or below, but significantly reduces it when the content reaches 20%. According to the research [16], SB is initially uniformly dispersed in the mortar. With the progress of cement hydration and decrease in water, SB gradually aggregates on the surface of the hydration products and they interweave with each other to form films which can wrap the cement inside. As a result, SB improves the steric hindrance of cement, hindering the hydration of cement.

### 3.5. Discussion

This paper explores the water transport properties of mortar, specifically focusing on waterproofness, impermeability, and water vapor transmission property. The results indicate that the pores in mortar are the primary factors influencing these three properties. Therefore, this section summarizes the impact of different modifiers on these properties, discusses the relationship between different modifiers, pore characteristics, and water transport properties, and explores how to balance both waterproofness and the water vapor transmission property of mortar.

The properties of the three modifications with good performance are shown in Table 8. Mechanical property is the basis for applying mortar. So, flexural strength, compressive strength, and tensile bond strength are tested. The results show that their mechanical property can meet the demands of practical applications, though some of them have decreased mechanical properties. The comparison of Section 3.1, Section 3.2 and Section 3.3 shows that modifying mortar with 20% SB results in excellent waterproofness with a 48-hour CWA rate of 8% compared to the comparison sample, the same impermeability as the comparison sample, and significantly enhanced water vapor transmission property with a 22% increase compared to the comparison sample. Compared to the comparison sample, mortar modified with 2.5% KH570 provides excellent water vapor transmission property and excellent waterproofness, but significantly reduces impermeability. Mortar modified with 2% XYPEX significantly increases its impermeability and enhances its waterproofness, but decreases its water vapor transmission property. Therefore, when optimizing waterproof mortar for exterior building walls with lower requirements for impermeability, mortar modified with either 20% SB or 2.5% KH570 can be chosen based on the requirements for water vapor transmission property and waterproofness.

**Figure 16:**
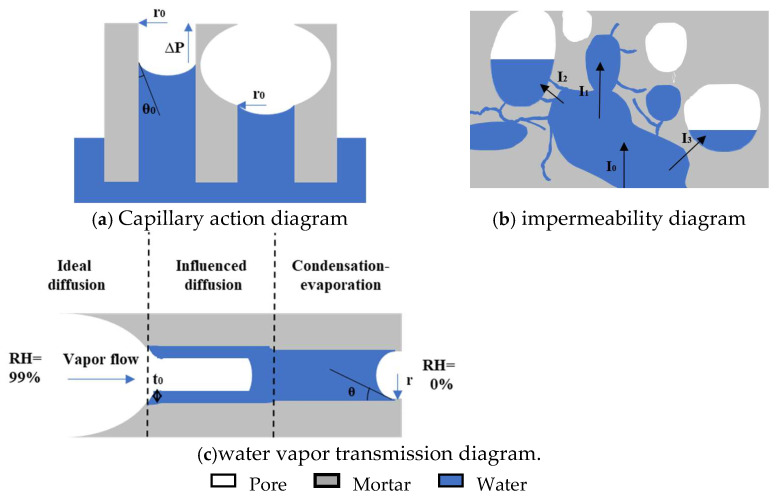
(**a**) Capillary action diagram; (**b**) impermeability diagram; (**c**) water vapor transmission diagram.

The CWA shows the waterproofness of mortar, and its main influencing factors include the porosity and pore size distribution of mortar, as well as the hydrophobicity and connectivity of pores, according to a study [37] and the analysis mentioned above. In CWA tests, the primary driving force for water transport is capillary action. As shown in Figure 16, under capillary action, water is transported from the liquid surface into the interior of the mortar along the pores, and the capillary force can be calculated by Equation (3). According to the equation, the smaller pore diameters result in enhanced capillary action, while larger water contact angles weaken capillary action. Therefore, reducing capillary porosity, optimizing pore size distribution, and adjusting water contact angles can all diminish capillary action. Furthermore, when pores are cut by larger air pores, capillary action is disrupted, and water transport caused by capillary action is weakened. Consequently, decreasing the connectivity of capillary pores can also lower CWA. XYPEX promotes cement hydration within pores, generating hydration products that block pores, thereby reducing the porosity of mortar and decreasing the number of both small and large capillary pores. These factors subsequently weaken capillary action and reduce CWA. KH570 hydrolyzes into silanol and methanol, where silanol undergoes self-condensation and condensation with –OH on the surface of hydration products, forming a hydrophobic layer on the pore surface [34]. The results indicate that KH570 increases the water contact angle of mortar, imparting hydrophobicity and significantly reducing CWA. However, excessive KH570 content slows cement hydration, decreasing the water contact angle of the modified mortar and losing its hydrophobicity. This is because too much condensed silanol increases the steric hindrance of unhydrated cement particles, hindering the formation of C–S–H gel and making it more difficult to form an effective hydrophobic layer on the surface. Additionally, KH570 has an air-entraining effect, primarily introducing large capillary pores ranging from 0.1 μm to 5 μm while reducing gel pores. Therefore, at higher KH570 contents, the increase in capillary pores becomes the dominant factor, substantially increasing the CWA of mortar. SB also has an air-entraining effect, mainly introducing air pores but reducing gel pores. At high contents, the air pores are regularly shaped and relatively isolated, with the formation of a complete and relatively continuous polymer film within the mortar. This film can block capillary pores and gel pores, reducing capillary action and thus significantly decreasing CWA.

The primary factors influencing impermeability are the porosity and pore connectivity of mortar. As shown in Figure 16, in impermeability tests, the main driving force for water transport is the externally applied pressure in the I_0_ direction. Under this external pressure, water first fills the pores connected to the outside. Once the pores are nearly filled with water, it will then be transported in the I_1_, I_2_, and I_3_ under the action of external pressure. Among them, I_1_ represents connected macropores, I_2_ represents connected capillary pores, and I_3_ represents a mortar layer with essentially no pores. At this point, due to steric hindrance, the resistance encountered follows the order I_1_ < I_2_ < I_3_. Therefore, reducing porosity and pore connectivity can increase the resistance to water intrusion under externally applied pressure, thereby enhancing the impermeability pressure of mortar. XYPEX reduces porosity and increases the compactness of mortar by generating hydration products, enabling it to resist water intrusion at higher pressures and exhibiting excellent impermeability. KH570 introduces a large number of pores, significantly increasing the porosity of mortar and reducing its compactness, thereby decreasing the ability to resist water intrusion of modified mortar. SB introduces a large number of air pores, causing it to nearly lose its impermeability at low contents. However, at high contents, SB forms a film effectively, which can block and seal the pores of the mortar. As a result, pore connectivity is greatly reduced, leading to a substantial recovery of the impermeability of the modified mortar.

The main factors influencing water vapor transmission property include porosity, pore size distribution, pore hydrophobicity, and pore connectivity. As shown in Figure 16, there are two primary forms of water vapor transmission through mortar [1]: water vapor transmission and capillary condensation-evaporation transport. Water vapor transmission can occur through ideal diffusion and transport influenced by surface adsorption depending on the pore size, with ideal diffusion being the fastest and capillary condensation-evaporation transport being the slowest. Therefore, larger pore sizes and higher porosity promote water vapor transmission, thereby enhancing water vapor transmission property, consistent with the conclusion of the study [38]. At a certain temperature and humidity conditions, pores smaller than a certain diameter will undergo capillary condensation, while pores larger than this diameter will not. According to the Kelvin equation, the critical radius for capillary condensation is determined by Equation (4). From this equation, it can be seen that when temperature and humidity are constant, a larger water contact angle results in a smaller critical radius, making capillary condensation less likely to occur and facilitating water vapor transmission through diffusion, which enhances water vapor transmission property. If pores are isolated, the water vapor transmission path is cut off, reducing vapor permeability. XYPEX reduces porosity by generating hydration products to block pores, increasing the number of smaller gel pores and decreasing the mode pore diameter, which decreases ideal diffusion and increases capillary condensation-evaporation transport. Consequently, it adsorbs a larger amount of water in a steady state and exhibits a lower water vapor permeability. KH570 introduces a large number of pores with larger diameters, increasing porosity and significantly increasing the mode pore diameter. Additionally, at low contents, KH570-modified mortar exhibits hydrophobicity, which can substantially reduce the critical radius for capillary condensation, enhance ideal diffusion, and thus improve the mortar’s water vapor transmission property, resulting in a higher water vapor permeability. SB introduces a large number of pores with larger diameters, increasing porosity and the mode pore diameter, which increases the ideal diffusion of vapor. However, when the SB content reaches 20%, due to its effective film-forming and sealing properties, pore connectivity is significantly reduced, hindering vapor diffusion and decreasing the water vapor transmission property of mortar, decreasing the water vapor permeability. Therefore, to have both excellent waterproofness and air permeability, admixtures similar to KH570 that have hydrophobic modification and air-entraining effects can be used to increase mortar porosity and hydrophobicity. This can enhance water vapor transmission property while inhibiting capillary action and reducing CWA.

(3) $$∆P=\frac{2\sigma cos\theta_{0}}{r_{0}}$$

∆P—Pressure caused by capillary action, Pa;

σ—Surface tension of water, N/m;

$\theta_{0}$—Contact angle between mortar and water, rad;

$r_{0}$—Pore radius, m.

(4) $$r=t_{0}-\frac{2M\sigma cos\theta }{\rho_{w}RTlnRH}$$

r—Critical pore radius for capillary condensation, m;

$t_{0}$—Adsorption layer thickness, m;

M—Molar mass of water, g/mol;

σ—Surface tension of water, N/m;

θ—Contact angle between mortar and water, rad;

$\rho_{w}$—Density of water, kg/m^3^;

R—Gas constant, J/(mol·K);

T—Temperature, K;

RH—Relative humidity, %.

## 4. Conclusions and Future Work

### 4.1. Conclusions

This paper explores the waterproofness, impermeability, and water vapor transmission property of three types of modified mortars at different modifier contents. The effects on these properties are analyzed. Furthermore, the factors affecting waterproofness, impermeability, and water vapor transmission property are discussed, and pore properties are used as a bridge to analyze how the three modifiers impact the waterproofness, impermeability, and water vapor transmission property of mortar. Lastly, the influencing factors and mechanisms of water transport are summarized, and the ways to balance waterproofness and air permeability are explored, providing a primary theoretical reference for the control of mortar’s waterproofness, impermeability, and water vapor transmission.

(1) XYPEX enhances the waterproofness of mortar to a certain extent and significantly improves its impermeability by promoting the formation of hydration products within the mortar pores, which reduces the porosity of air pores and capillary pores. However, it also decreases the water vapor transmission property of mortar, and the effect becomes more pronounced at higher contents

(2) At contents of 1.25% and 2.5%, KH570 introduces air pores into the mortar while imparting hydrophobicity, thereby improving waterproofness, reducing impermeability, and significantly enhancing water vapor transmission property. However, at contents of 3.75% and 5%, the KH570-modified mortar exhibits poor hydrophobic modification and introduces more capillary pores, resulting in poor waterproofness and impermeability.

(3) When SB is added at contents of 15% and 20%, it introduces regularly shaped air pores and forms a polymer film that can seal smaller capillary pores and gel pores, reducing the capillary pore porosity. Consequently, the mortar exhibits excellent waterproofness and water vapor transmission property. At a 20% SB content, the introduced air pores have low connectivity, allowing the mortar to maintain its impermeability while also meeting higher requirements for waterproofness and water vapor transmission property.

(4) By analyzing the relationship between pores and water transport, the following influencing factors and mechanisms of water transport have been discussed: Reducing capillary pore porosity, enhancing pore hydrophobicity, and decreasing pore connectivity can weaken or block capillary action, thereby reducing the CWA. Decreasing porosity and pore connectivity can reduce the spatial hindrance for the transmission of additional pressure water within the mortar, improving its impermeability. Adjusting the pore size distribution and hydrophobicity of the mortar can change the method of water vapor transmission property, and increasing the amount of vapor diffusion with higher transmission rates to enhance water vapor transmission property. Increasing porosity and pore connectivity can also significantly increase the transmission pathways for vapor diffusion, further improving the mortar’s water vapor transmission property.

(5) Mortar modified by 2.5% KH570 has excellent waterproofness and water vapor transmission property, but poor impermeability; mortar modified by 20% SB has the best waterproofness and relatively excellent water vapor transmission property, and the impermeability is basically the same as that of the comparison sample.

### 4.2. Future Work

(1) The existing work has only focused on the service effect under ideal conditions, which is far different from real-world applications. So, long-term performance of modified mortar at environmental stressors needs to be studied. Only with durability and real-condition testing can this theory be used to guide real-world applications. 

(2) The comparison sample for modified mortar is a sample without any modification. Though it is obvious to study how modifiers influence the mortar, the strength of the modifiers compared to commercially available waterproofing mortars is not clear. So, some extra samples that are commercially available will be tested.

## Figures and Tables

**Table 1:** Chemical composition of the cement (wt.%).

CaO	SiO_2_	Al_2_O_3_	SO_3_	Fe_2_O_3_	MgO	K_2_O	TiO_2_	SrO	Na_2_O	SUM
62.60	19.80	4.63	3.87	3.49	1.64	0.97	0.27	0.18	0.17	97.62

**Table 2:** Residual screening of sand.

Residual Size (mm)	2.36	1.18	0.6	0.3	0.15	<0.15
Percentage retained (%)	0	24.48	27.55	31.19	11.16	5.02
Cumulative percentage retained (%)	0	24.48	52.03	83.22	94.98	100

**Table 3:** Chemical composition of the XYPEX (wt.%).

CaO	SiO_2_	MgO	Al_2_O_3_	SO_3_	Fe_2_O_3_	Na_2_O	K_2_O	SUM
58.53	16.10	13.30	4.36	2.79	1.84	1.29	0.44	98.65

**Table 4:** The abbreviations of different groups.

Abbreviation	Cement/Sand (Only Mortar)	Water/Cement	XYPEX (wt.%)	KH570 (wt.%)	SB (wt.%)
C	1:3	0.500			
X1		0.494	0.5		
X2		0.490	1.0		
X3		0.487	1.5		
X4		0.483	2.0		
K1		0.480		1.25	
K2		0.530		2.50	
K3		0.570		3.75	
K4		0.600		5.00	
S1		0.463			5
S2		0.421			10
S3		0.380			15
S4		0.320			20

**Table 5:** Capillary water absorption rate at each stage (kg·m^−2^·h ^−1/2^) and 48 h capillary water absorption.

	I	II	III	48 h Capillary Water Absorption (kg/m^2^)
C	0.47	0.16	0.12	1.50
X1	0.48	0.15	0.10	1.41
X2	0.37	0.14	0.10	1.24
X3	0.36	0.12	0.09	1.16
X4	0.35	0.12	0.09	1.12
K1	0.36	0.16	0.10	1.35
K2	0.26	0.12	0.07	0.98
K3	0.42	0.28	0.23	2.13
K4	0.42	0.34	0.32	2.53
S1	0.89	0.26	0.18	2.61
S2	0.52	0.18	0.13	1.67
S3	0.12	0.08	0.08	0.61
S4	0.04	0.01	0.01	0.12

**Table 6:** Characteristic pore size and porosity of modified mortars.

Group	Average Pore Diameter	Medium Pore Diameter	Mode Diameter	Porosity (%)
C	32.6 nm	63.9 nm	50.4 nm	12.4
X2	30.5 nm	61.4 nm	50.4 nm	12.1
X4	29.4 nm	58.0 nm	50.3 nm	12.0
K2	71.7 nm	601.3 nm	2.5 μm	19.5
K4	72.1 nm	959.0 nm	1.1 μm	19.4
S2	162.4 nm	92.3 μm	181.1 μm	28.9
S3	156.0 nm	85.7 μm	180.9 μm	27.4
S4	127.6 nm	51.2 μm	90.5 μm	24.7

**Table 7:** Percentage of each form of moisture in different modified mortars (%).

Group	Gel Pores Water	Capillary Water	Free Water
C	72	18	10
X2	68	14	8
X4	72	26	2
K2	73	22	5
K4	68	25	7
S2	65	22	13
S3	64	26	10
S4	58	31	11

**Table 8:** Properties of three modifications with good performance.

Group	48 h CWA (kg·m^2^)	Impermeability Stress (MPa)	Water Vapor Permeability (g·MPa^−1^·h^−1^·m^−1^)	28 d Flexural Strength (MPa)	28 d Compressive Strength (MPa)	28 d Tensile Bond Strength (MPa)
C	1.50	1.4	10.7	11.2	58.3	1.79
X4	1.12	>1.5	8.1	9.4	59.8	1.62
K2	0.98	0.6	22.6	9.2	45.8	1.87
S4	0.12	1.4	16.7	12.4	42.3	4.53

## Data Availability

The original contributions presented in this study are included in the article. Further inquiries can be directed to the corresponding author(s).
